# Three-Year Apical Outcomes in Teeth with Apical Periodontitis Following Primary Endodontic Treatment or Nonsurgical Retreatment: A Retrospective Clinical Study

**DOI:** 10.3390/jcm15187256

**Published:** 2026-09-18

**Authors:** Cristian Marius Băcanu, Cristian Niky Cumpătă, Alexandru Dan Popescu, Oana Andreea Diaconu, Carina Alexandra Bănică, Mihaela Jana Ţuculină, Denisa Alexandra Al Share, Mihaela Răescu, Mihaela Ionescu, Lelia Mihaela Gheorghiță

**Affiliations:** 1 Doctoral School, University of Medicine and Pharmacy of Craiova, 200349 Craiova, Romania; bacanucristian@yahoo.com; 2 Department of Oral and Maxillofacial Surgery, Faculty of Dental Medicine, Titu Maiorescu University Bucharest, 031593 Bucharest, Romania; 3 Department of Endodontics, Dental Medicine Research Center, Faculty of Dentistry, University of Medicine and Pharmacy of Craiova, 200349 Craiova, Romania; alexandrudanpopescu20@gmail.com (A.D.P.); oanamihailescu76@yahoo.com (O.A.D.); karinabanica@yahoo.com (C.A.B.); leliagheorghita@yahoo.com (L.M.G.); 4 Dentist, Dental Clinic, 1 Burebista Blvd., 031106 Bucharest, Romania; 5 Department of Oro-Dental Prevention, Faculty of Dental Medicine, “Titu Maiorescu” University Bucharest, 031593 Bucharest, Romania; mihaela.raescu@gmail.com; 6 Department of Medical Informatics and Biostatistics, Faculty of Dentistry, University of Medicine and Pharmacy of Craiova, 200349 Craiova, Romania; mihaela.ionescu@umfcv.ro

**Keywords:** periapical healing, apical periodontitis, endodontic retreatment, NiTi instrumentation, nickel–titanium, root canal treatment

## Abstract

**Background/Objectives**: This retrospective observational study assessed 36-month final apical status after primary endodontic treatment and nonsurgical retreatment performed using different nickel–titanium (NiTi) instrumentation systems and explored its association with available clinical and morphological variables. **Methods**: The study included 89 permanent teeth from 69 patients treated between January 2022 and January 2023. Teeth with periapical lesions and complete baseline and 36-month clinical and radiographic records were evaluated using a stringent binary endpoint in which complete healing required the absence of clinical signs or symptoms and residual apical radiolucency. Associations were examined primarily using univariable logistic generalized estimating equation (GEE) models accounting for clustering of teeth within patients, while Pearson’s chi-square tests and Cohen’s w were retained as supplementary unadjusted analyses. Because CBCT/Diagnocat information could contribute to final outcome classification when available, Diagnocat-based CBCT availability was not analyzed as an explanatory variable. **Results**: Complete clinico-radiographic healing at 36 months was observed in 61 teeth (68.54%). When reported separately by treatment modality, complete healing was observed in 12 of 29 primary-treatment cases (41.38%) and in 49 of 60 nonsurgical retreatment cases (81.67%). Because treatment allocation was non-random and the groups differed in case characteristics, this contrast was considered exploratory rather than an estimate of comparative treatment effectiveness. Exploratory analyses showed no evidence of an association with the NiTi instrumentation system or type of NiTi motion. **Conclusions**: Subgroup comparisons were exploratory, unadjusted for multiple testing, and should not be interpreted as evidence of independent prognostic effects. Final apical status should therefore be interpreted within the broader context of treatment indication, tooth morphology, the method of outcome ascertainment, and the overall therapeutic protocol rather than attributed to a specific NiTi system.

## 1. Introduction

Apical periodontitis represents the inflammatory expression of persistent endodontic infection and remains one of the main indications for primary endodontic treatment and nonsurgical endodontic retreatment. In current clinical practice, the therapeutic objective is not limited to intracanal infection control, but also aims to achieve periapical healing, defined by the absence of clinical signs and symptoms and by the resolution or absence of radiographic signs of periapical pathology. The ESE S3 clinical guideline published in 2023, together with the 2024 update commentary, supports a combined clinical and imaging-based assessment of endodontic treatment outcomes [1,2].

Clinical endodontic success is generally characterized by the retention of a functional and asymptomatic tooth, without pain, tenderness to percussion, swelling, or sinus tract, together with the absence or progressive resolution of radiographic signs of apical disease. However, outcome definitions vary among studies, particularly regarding whether residual but decreasing apical radiolucency is accepted as favorable healing.

The outcome of endodontic treatment is multifactorial. Classical clinical studies and systematic reviews have shown that preoperative pulpal and periapical status, lesion size, the ability to instrument the canal to the working length, the quality of root canal filling, and the quality of the coronal restoration may influence prognosis [3,4]. This prognostic framework has also been supported by more recent clinical literature addressing lesion characteristics, canal anatomy, and technical treatment quality [5,6]. In parallel, experimental evidence indicates that different irrigation protocols can influence root canal cleaning and irrigant penetration, supporting the biological relevance of the disinfection strategy [7].

In this context, mechanical root canal instrumentation represents a central stage of treatment, because it influences debridement, canal shaping, and irrigation efficacy. Available evidence suggests that contemporary instrumentation techniques may be associated with more favorable outcomes than conventional stainless-steel hand instrumentation, but does not allow a consistent healing advantage to be attributed to a specific modern NiTi system [8]. At the same time, the biological outcome of endodontic treatment does not depend exclusively on instrumentation, since root filling techniques and materials, including sealer type, may influence outcome assessment in teeth with apical periodontitis [9,10]. For nonsurgical endodontic retreatment, the reported outcomes are favorable but variable, depending on case selection, assessment criteria, and imaging follow-up method [11,12].

Against this background, the present study was designed to provide a 36-month clinico-radiographic evaluation of final apical status after primary endodontic treatment and nonsurgical endodontic retreatment performed using different NiTi instrumentation systems. The study also aimed to analyze the distribution of healed/not healed final apical status in relation to the variables available in the retrospective records, including the NiTi system used, the type of NiTi motion, tooth type, number of roots, and treatment modality. The working hypothesis was that final apical status would not be explained by the NiTi system alone, but would vary according to treatment modality and the clinical and morphological characteristics of the included cases. Therefore, the analysis of NiTi systems was performed within a broader clinical and morphological framework, in the context of the absence of random allocation.

## 2. Materials and Methods

### 2.1. Study Design and Setting

The present study was designed as a retrospective observational study based on the review of pre-existing clinical records and imaging documentation. The records screened for eligibility corresponded to patients treated in routine clinical practice between January 2022 and January 2023 in the Endodontics Clinic of the Faculty of Dentistry, University of Medicine and Pharmacy of Craiova. The 36-month clinical and radiographic follow-up visits were performed as part of routine clinical care and were completed between January 2025 and January 2026. The study protocol, including retrospective record screening, data extraction, anonymization, database preparation, and statistical analysis, was approved by the Ethics Committee of the University of Medicine and Pharmacy of Craiova, approval no. 295, dated 10 July 2025. All research-related procedures were performed after ethics approval had been obtained. No prospective enrollment, study-related intervention, or additional clinical procedure was performed before ethics approval. Reporting followed the STROBE recommendations for observational studies.

### 2.2. Participants

Eligibility was assessed at the tooth level. Teeth were included only when all of the following criteria were fulfilled: (1) permanent tooth; (2) clinically and radiographically confirmed apical periodontitis/periapical lesion of endodontic origin; (3) treatment by either primary endodontic treatment or nonsurgical endodontic retreatment during the predefined study period; (4) availability of baseline clinical data; (5) availability of baseline imaging documentation; (6) complete documentation of the endodontic treatment performed, including identification of the NiTi instrumentation system used; and (7) availability of a documented 36-month post-treatment clinical and radiographic reassessment.

Teeth were excluded if any of the following applied: (1) incomplete clinical records that prevented reliable extraction of the required study variables; (2) absence of baseline imaging documentation; (3) absence of a documented 36-month clinical and radiographic follow-up; (4) absence of information identifying the NiTi instrumentation system used; or (5) treatment involving endodontic surgery. Only nonsurgically treated teeth were therefore eligible for the present analysis. Records for which patient consent for the use of clinical data was unavailable were not entered into the eligibility assessment.

**Figure 1:**
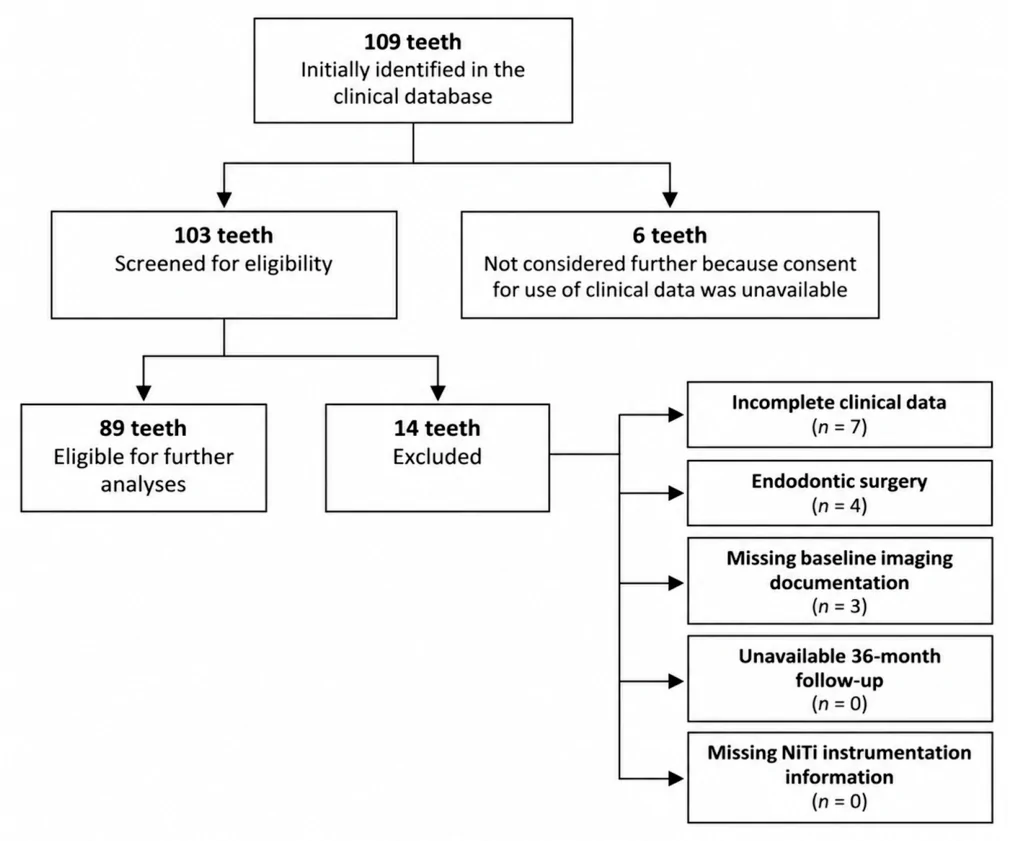
Flow diagram of case identification, eligibility screening, and inclusion.

During the study period, 109 consecutive teeth with apical periodontitis were identified in the clinical database and constituted the initial pool of potentially eligible cases. Six teeth were not considered further because consent for the use of clinical data was unavailable. Of the remaining 103 teeth screened for eligibility, 14 were excluded: seven because of incomplete clinical data, four because they had undergone endodontic surgery, and three because baseline imaging documentation was unavailable. No teeth were excluded specifically because the 36-month clinical and radiographic follow-up was unavailable or because information on the NiTi instrumentation system was missing. The final analyzed sample comprised 89 teeth from 69 patients (Figure 1).

The unit of analysis was the tooth, with teeth nested within patients. Of the 69 patients, 52 contributed one tooth, 14 contributed two teeth, and 3 contributed three teeth; cluster size ranged from 1 to 3 teeth per patient. Within-patient correlation in the binary 36-month final apical status was quantified using an intercept-only mixed-effects logistic regression model with a patient-specific random intercept. The latent-scale intraclass correlation coefficient was 0.15 (95% CI: 0.051–0.730), indicating considerable uncertainty regarding the magnitude of within-patient correlation. Therefore, patient-level clustering was accounted for in the subsequent principal inferential analyses.

### 2.3. Variables

The primary outcome variable was the final apical status at 36 months, dichotomously classified as healed or not healed according to the combined clinical and radiographic criteria recommended for endodontic outcome assessment [1,2]. A tooth was classified as healed when, at the 36-month reassessment, there was no pain on occlusal/vertical percussion, no local clinical signs such as sinus tract or inflammatory swelling, and no radiographic evidence of apical radiolucency. Teeth that did not meet all these criteria were classified as not healed. Because changes in lesion dimensions were not consistently documented, incomplete radiographic healing could not be distinguished from persistent disease within the not-healed category; accordingly, this category should not be interpreted as synonymous with persistent disease or treatment failure. Because primary endodontic treatment and nonsurgical retreatment represent clinically distinct treatment contexts, modality-specific healing proportions were reported, while the overall 89-tooth healing estimate was considered descriptive only and was not interpreted as a common prognostic estimate for the two treatment categories.

The radiographic component of this classification was based on the imaging documentation available during routine care. Periapical radiographs were available for all included teeth; however, when CBCT/Diagnocat documentation was also available, these additional findings could contribute to the final radiographic assessment. Consequently, outcome ascertainment was not based on an identical imaging modality in all teeth.

The following variables were recorded: patient gender, tooth quadrant, tooth type, number of roots, NiTi instrumentation system, and treatment modality. Gender was a patient-level characteristic; when descriptive data were presented at the tooth level, each tooth was assigned the gender of the corresponding patient. Consequently, tooth-level gender counts do not represent independent patient counts. The availability of Diagnocat-based CBCT documentation was recorded separately as an imaging-ascertainment characteristic and was not treated as an explanatory variable. Tooth type was operationalized as anterior versus posterior teeth. Number of roots was classified as single-rooted versus multi-rooted and was used as the available retrospective morphological variable. The number of root canals was not included as a separate variable because it was not consistently documented in the contemporaneous treatment records. Canal count was not retrospectively inferred from periapical radiographs because conventional two-dimensional imaging does not consistently depict the complete root canal anatomy, while CBCT was not uniformly available across the cohort [13]. Such retrospective reconstruction from non-uniform imaging sources could therefore have introduced differential ascertainment of this variable.

The present analysis was not designed as a comprehensive prognostic-factor study. Age, systemic conditions, smoking status, periodontal status, baseline lesion dimensions or periapical index scores, pulpal diagnosis, number of root canals, baseline technical quality, root filling quality, coronal restoration quality, and procedural complications were not included because these variables were either not consistently documented or had not been recorded in a sufficiently standardized form for reliable retrospective analysis. Because these variables were unavailable or insufficiently standardized, no risk-adjusted comparison between primary treatment and nonsurgical retreatment was attempted. Differences between treatment modalities were therefore interpreted only as descriptive and exploratory subgroup findings. Clinician-related variability was not evaluated because all endodontic procedures were performed by the same experienced endodontic specialist. Given the limited number of not-healed outcomes, inclusion of a substantially larger number of explanatory parameters in a prognostic model of the 36-month healing outcome would have resulted in an excessively overparameterized model.

To address the type of NiTi motion, an additional exploratory variable was derived from the recorded instrumentation system. RECIPROC blue R25 was classified as a reciprocating NiTi system, whereas ProTaper Ultimate and VDW.ROTATE were classified as continuous-rotation NiTi systems.

### 2.4. Data Sources and Clinical/Radiological Assessment

Data were extracted from the clinical records and imaging documentation available for each case. Clinical assessment included evaluation of pain on occlusal/vertical percussion and identification of local clinical signs, particularly the presence of a sinus tract or inflammatory swelling. Radiographic assessment focused on the presence or absence of apical radiolucency.

Baseline and 36-month follow-up periapical radiographs were acquired using a CS 2200 intraoral X-ray unit (Carestream Dental LLC, Atlanta, GA, USA) and an RVG 6200 digital intraoral sensor (Carestream Dental LLC, Atlanta, GA, USA). Radiographs were obtained using the paralleling technique with a sensor-positioning device. Whenever technically feasible, the same positioning system and comparable horizontal and vertical projection geometry were used at baseline and follow-up. Exposure settings were selected using the anatomical presets of the X-ray unit according to tooth region, patient anatomy, and the digital receptor. The exact exposure time used for each individual radiograph was not consistently retained in the retrospective records. Digital images were displayed and reviewed using the manufacturer’s imaging software under routine clinical viewing conditions.

When available in the clinical record, imaging documentation generated with the AI-assisted software Diagnocat (version 2.0, Canada) was used as an adjunct to the conventional clinical and radiographic assessment of periapical lesions. The Diagnocat-based imaging documentation used in the present study was derived from cone-beam computed tomography (CBCT) examinations obtained during routine clinical care at the treating clinician’s discretion rather than according to a predefined study protocol. The specific clinical indication for CBCT examination was not consistently recorded and could not be reliably reconstructed for all cases. The AI-assisted assessment was not considered a therapeutic intervention or a stand-alone diagnostic criterion. However, when CBCT/Diagnocat documentation was available, information from this additional imaging assessment could contribute to the radiographic component of the final healed/not-healed classification. Diagnocat availability was therefore recorded descriptively but was not analyzed as an explanatory variable for final apical status. Clinical signs, periapical radiographic findings, and the documented imaging assessment were reviewed retrospectively from the existing patient records.

When CBCT examinations were available, selected clinically complex cases were additionally reviewed by an oral and maxillofacial surgeon as part of routine clinical care. This additional review did not modify the predefined healed/not-healed criteria, although CBCT/Diagnocat findings could contribute to determining whether residual apical pathology was present.

The final apical status was assessed retrospectively from the clinical and imaging documentation available in the patient record. Although the same operational healed/not-healed criteria were applied, the imaging information available for outcome ascertainment was not identical across all teeth because CBCT/Diagnocat findings could contribute to the assessment when present. A periapical-radiograph-only 36-month endpoint could not be retrospectively reconstructed for all teeth independently of the CBCT/Diagnocat findings. Therefore, the primary 36-month outcome should be regarded as a retrospective clinico-imaging endpoint based on the documentation available for each tooth, rather than as a uniform periapical-radiograph-only endpoint. No independent blinded radiographic reassessment was performed, and interobserver agreement was not calculated, because the study was based on retrospective clinical records and on the imaging documentation available in the patients’ files.

Post-treatment follow-up was performed according to the clinical recall protocol. For the present analysis, only teeth with a documented 36-month clinical and radiographic reassessment were included. The 36-month time point was selected because it represented a uniform long-term clinical and radiographic reassessment available for all included teeth. The ESE S3-level clinical practice guideline and its subsequent update informed the use of combined clinical and imaging criteria for outcome assessment [1,2]; the 36-month interval itself reflects the standardized follow-up point available in this retrospective cohort rather than a guideline-mandated interval.

At the original follow-up visits, periapical radiographs were interpreted by the treating endodontist, an endodontic specialist with 20 years of clinical experience. Cases with uncertain radiographic findings were reviewed by a senior endodontist before the final assessment was entered into the clinical record. Both examiner assessments were performed as part of the routine clinical workflow using the same predefined healed/not-healed criteria. No study-specific examiner calibration, independent blinded reassessment, or formal interobserver agreement analysis was performed because the study relied on assessments documented during routine clinical care.

No ordinal radiographic lesion-scoring system was applied. Radiographic status was recorded dichotomously as the absence or presence of apical radiolucency. A tooth was classified as healed only when no residual apical radiolucency was present at the 36-month assessment. Any residual apical radiolucency resulted in classification as not healed, irrespective of whether its dimensions appeared reduced compared with baseline. Lesion dimensions were not measured quantitatively because sufficiently standardized measurements were not consistently available in the retrospective records.

### 2.5. Bias

To reduce the risk of information bias, only cases with sufficient clinical and imaging documentation to establish the initial diagnosis and final apical status were included in the analysis. To limit sample heterogeneity, only nonsurgically treated cases were included, and the outcome was defined according to predefined clinical and radiographic criteria. Potential sources of bias inherent to the retrospective design, including case selection based on documentation availability and variability in the quality of clinical records, were considered when interpreting the results.

An additional source of information bias was the non-uniform imaging ascertainment of the 36-month outcome. Because CBCT/Diagnocat documentation was available only for a subset of teeth and could reveal persistent apical pathology not evident on periapical radiographs, differential detection bias cannot be excluded. For this reason, Diagnocat availability was not evaluated as an explanatory factor for final apical status.

Because inclusion in the final analysis required complete baseline and treatment documentation, selection bias related to record completeness cannot be excluded. The available retrospective information for excluded cases was insufficiently complete and standardized to support a meaningful comparison with the included cohort.

### 2.6. Study Size

The initial screening pool comprised all 109 consecutive teeth with apical periodontitis identified in the clinical database during the predefined treatment period. The final study size was determined after application of the consent requirement and the predefined eligibility criteria, including availability of the required baseline, treatment, and 36-month follow-up documentation. The detailed screening process and reasons for exclusion are presented in Figure 1.

An a priori sample-size calculation was not used to determine the study size because the study was designed to include all consecutive eligible cases identified during the predefined treatment period, rather than to recruit a prespecified number of observations. Accordingly, the available sample size was determined by the number of eligible cases meeting the predefined inclusion criteria and having the required 36-month follow-up documentation. Post hoc achieved power was estimated for the supplementary chi-square comparisons using the observed Cohen’s w effect sizes, a two-sided α level of 0.05, *N* = 89, and the noncentral chi-square distribution. Achieved power was 0.970 for the association between treatment modality and final apical status, 0.272 for NiTi instrumentation system, 0.076 for quadrant, 0.963 for tooth type, 0.728 for root morphology, and 0.062 for type of NiTi motion. These calculations indicated high achieved power for some of the larger observed associations but limited power for weaker associations. Because achieved power is conditional on the observed effect size and these calculations do not account for within-patient clustering, they were interpreted descriptively and did not replace the cluster-adjusted GEE estimates and confidence intervals.

### 2.7. Endodontic Treatment and Follow-Up

The included teeth underwent either primary endodontic treatment or nonsurgical endodontic retreatment. Primary endodontic treatment was defined as the initial root canal treatment of a tooth that had not previously undergone endodontic therapy. It included access cavity preparation, canal identification, chemomechanical instrumentation, disinfection, and root canal obturation. Nonsurgical endodontic retreatment was defined as the reopening of a previously treated tooth, removal of the existing root canal filling material, reinstrumentation and disinfection of the canal system, and subsequent root canal obturation, without a surgical endodontic procedure. Treatment modality was selected according to the previous endodontic status and clinical indication of each tooth.

All procedures were performed according to the routine endodontic protocol of the clinic by a single endodontic specialist with 20 years of clinical experience. Teeth were isolated with a rubber dam and treated under an operating microscope. Working length was determined using an electronic apex locator (J. Morita Mfg. Corp., Kyoto, Japan) and confirmed by periapical radiography. Canal patency and glide-path preparation were performed before engine-driven instrumentation according to the clinical requirements of each canal. However, the retrospective records did not consistently document the manual file sizes or the specific glide-path sequence used in each individual case. The instrumentation systems used were RECIPROC blue R25 and VDW.ROTATE (VDW GmbH, Munich, Germany) and ProTaper Ultimate (Dentsply Sirona, Ballaigues, Switzerland). RECIPROC blue R25 was used in reciprocating motion with the dedicated “RECIPROC all” motor preset. The RECIPROC blue R25 instrument has an ISO tip size of 25 and a regressive taper of 0.08 over the first apical millimeters. VDW.ROTATE was used in continuous rotation according to the manufacturer’s recommended sequence, generally comprising 15/0.04 and 20/0.05 instruments, followed by 25/0.06 for most canals or 25/0.04 for narrow and markedly curved canals. The recommended rotational speed was 300–400 rpm, with torque adjusted according to instrument size.

ProTaper Ultimate (Dentsply Sirona, Ballaigues, Switzerland) was used in continuous rotation according to the manufacturer-recommended sequence. The sequence comprised the Slider 16/0.02v and Shaper 20/0.04v, followed by one or more finishing instruments selected according to canal anatomy and apical dimensions: F1 20/0.07v, F2 25/0.08v, F3 30/0.09v, FX 35/0.12v, or FXL 50/0.10v. The instruments were operated at 400 rpm and within the manufacturer-recommended torque range of 4.0–5.2 N·cm.

During instrumentation, the root canals were irrigated with 5% sodium hypochlorite. A 17% ethylenediaminetetraacetic acid (EDTA; Ultradent Products Inc., South Jordan, UT, USA) solution was used for smear-layer removal. Sterile 0.9% sodium chloride solution was used as an intermediate rinse between the different irrigating solutions to prevent their direct interaction. As a final adjunctive irrigant, 2% chlorhexidine (Consepsis; Ultradent Products Inc., South Jordan, UT, USA) was applied after an intermediate rinse with sterile 0.9% sodium chloride solution (B. Braun Melsungen AG, Melsungen, Germany) and drying of the root canals. The retrospective records did not consistently document the total irrigant volume, delivery needle characteristics, exposure time, or activation method for each tooth. Consequently, these case-level parameters could not be reliably reconstructed and are acknowledged as a limitation of the retrospective design.

In cases treated over multiple visits, 35% calcium hydroxide paste (UltraCal XS; Ultradent Products Inc., South Jordan, UT, USA) was used as an intracanal medicament, and the access cavity was temporarily sealed with glass ionomer cement (Fuji IX GP; GC Corporation, Tokyo, Japan). Cases requiring interappointment intracanal medication were therefore completed over at least two visits. The exact distribution of single-visit and multiple-visit treatments was not consistently available in the retrospective records. At the subsequent visit, the intracanal medicament was removed, and the canals were irrigated and dried before obturation. Root canals were obturated using warm vertical compaction of gutta-percha (ProTaper Ultimate Conform Fit; Maillefer Instruments Holding Sàrl, Ballaigues, Switzerland) in combination with AH Plus Bioceramic Sealer (Dentsply Sirona, Ballaigues, Switzerland). The specific definitive restorative material was not consistently recorded in the retrospective dataset and could not be reported reliably for every included tooth.

In nonsurgical retreatment cases, the existing root canal filling material was removed using ProTaper Universal Retreatment instruments (Dentsply Sirona, Ballaigues, Switzerland). The dedicated D1, D2, and D3 instruments were used sequentially, as clinically indicated, for removal of filling material from the coronal, middle, and apical thirds. A 10% citric acid solution (Citocid; AMMDENT, Mohali, India) was used as an adjunctive chelating irrigant. After removal of the previous filling material, canal patency and working length were re-established, and the canals were subsequently reshaped using the instrumentation system recorded as the study variable, disinfected, and obturated according to the same clinical protocol. The access cavity was subsequently restored with a definitive restorative material.

Because of the retrospective design, some case-level procedural details—including the exact glide-path sequence, final apical instrument used, irrigant volumes and exposure times, activation parameters, number of visits, and the specific definitive restorative material—were not consistently available for all teeth and could not be reliably reconstructed.

### 2.8. Statistical Analysis

Microsoft Excel 365 (Microsoft Corp., Redmond, WA, USA) was used for data organization, coding of categorical variables, and descriptive summaries. Supplementary contingency-table analyses were performed in SPSS version 30 (IBM Corp., Armonk, NY, USA), whereas the GEE analyses were performed in Python version 3.13.5 using statsmodels version 0.14.6. Post hoc achieved-power calculations for the supplementary chi-square analyses were performed in Python using SciPy version 1.17.0 and the noncentral chi-square distribution.

Because the unit of analysis was the tooth and some patients contributed more than one tooth, the principal inferential analyses accounted for within-patient clustering. Separate binary logistic generalized estimating equation (GEE) models were fitted using patient identification as the clustering variable, an independent working correlation structure, and robust sandwich standard errors. For analyses of the 36-month outcome, complete clinico-radiographic healing was coded as 1 and failure to meet the complete-healing criteria as 0. For analyses examining treatment modality as the dependent variable, nonsurgical retreatment was coded as 1 and primary endodontic treatment as 0. In the additional exploratory analysis in which NiTi instrumentation system was the dependent variable, its three nominal categories were analyzed using multinomial logistic regression with GEE, robust standard errors, and an exchangeable correlation structure. VDW.ROTATE was defined as the reference outcome category, generating two logit comparisons: RECIPROC blue R25 versus VDW.ROTATE and ProTaper Ultimate versus VDW.ROTATE. Because multiple teeth could belong to the same patient, patient identification was used as the clustering variable in this multinomial model, with robust standard errors. The multinomial model simultaneously included quadrant, tooth type, and root morphology as predictors. Q2, anterior teeth, and single-rooted morphology were used as the respective reference categories. Quadrant contributed three indicator coefficients, whereas tooth type and root morphology each contributed one coefficient, and each non-reference logit additionally included an intercept. Thus, six coefficients were specified for each non-reference logit, corresponding to 12 coefficients in total. Because of quasi-complete separation, some coefficients and the overall robust Wald test for the multicategory quadrant factor were non-estimable.

Binary predictors were indicator-coded using primary treatment, anterior tooth, single-rooted morphology, and reciprocating motion as the respective reference categories. In the binary GEE models, NiTi instrumentation system and quadrant were treated as nominal categorical predictors and entered using indicator variables. VDW.ROTATE was used as the reference category for NiTi instrumentation system and Q2 as the reference category for quadrant. Accordingly, binary predictors contributed one regression coefficient, the three-level NiTi factor contributed two coefficients, and the four-level quadrant factor contributed three coefficients; each model additionally included an intercept. In the binary GEE models, for multicategory predictors, an overall robust Wald chi-square test of the factor was evaluated before category-specific odds ratios, 95% confidence intervals, and nominal *p* values were considered.

The GEE models were regarded as the principal inferential analyses because they accounted for within-patient clustering; however, all subgroup comparisons remained exploratory and were not intended to establish independent prognostic effects.

Pearson’s chi-square tests were retained as supplementary unadjusted analyses of the corresponding contingency tables, and Cohen’s w was used to quantify effect size. Cohen’s w was calculated from the chi-square statistic and the total sample size. Effect sizes were interpreted as negligible for values <0.10, small for values from 0.10 to <0.30, moderate for values from 0.30 to <0.50, and large for values ≥0.50. Because some contingency tables included small expected cell counts, the corresponding results were interpreted cautiously and regarded as exploratory. No adjustment for multiple exploratory comparisons was applied. Accordingly, all reported *p* values are nominal and were interpreted descriptively as exploratory signals rather than as confirmatory evidence.

Diagnocat-based CBCT availability was not included in the association analyses because the additional imaging information could contribute to outcome ascertainment when available, creating a risk of differential detection bias.

## 3. Results

The final study sample comprised 89 teeth from 69 patients. Of these, 22 teeth (24.72%) were anterior teeth and 67 (75.28%) were posterior teeth. Forty-nine teeth (55.06%) were single-rooted and 40 (44.94%) were multi-rooted. The RECIPROC blue R25 system was used in 34 teeth (38.20%), ProTaper Ultimate in 13 teeth (14.61%), and VDW.ROTATE in 42 teeth (47.19%). According to the type of NiTi motion, 34 teeth (38.20%) were instrumented with a reciprocating system and 55 teeth (61.80%) with continuous-rotation systems (Table 1).

Diagnocat-based imaging documentation was available for 74 teeth (83.15%) and unavailable for 15 teeth (16.85%). Primary treatment was performed in 29 teeth (32.58%), whereas 60 teeth (67.42%) underwent nonsurgical retreatment. At the 36-month follow-up, 61 teeth (68.54%) met the predefined criteria for complete clinico-radiographic healing, whereas 28 teeth (31.46%) did not meet these criteria (Table 1). Among the 28 teeth that did not meet the complete-healing criteria, 10 were clinically asymptomatic but showed residual apical radiolucency, whereas 18 presented persistent clinical signs and/or symptoms at the 36-month assessment.

All included teeth had a documented 36-month clinical and radiographic follow-up. The final apical status was therefore assessed at a uniform 36-month time point for all teeth.

### 3.1. Final Apical Status Analysis

At the 36-month follow-up, 61 teeth (68.54%) met the predefined criteria for complete clinico-radiographic healing, whereas 28 teeth (31.46%) did not meet these criteria. Because changes in lesion dimensions were not recorded with sufficient standardization, the latter group could not be subdivided on the basis of radiographic lesion evolution into incomplete healing and persistent disease. Because some patients contributed more than one tooth, associations between final apical status and the explanatory variables were evaluated primarily using univariable GEE models accounting for patient-level clustering. Pearson’s chi-square tests and Cohen’s w were retained as supplementary unadjusted analyses. The results are presented in Table 2.

In the principal cluster-adjusted exploratory GEE analysis, nonsurgical retreatment showed higher odds of complete clinico-radiographic healing than primary treatment (OR = 6.205, 95% CI 2.088–18.439, nominal *p* = 0.001). Complete healing was observed in 12 of 29 teeth treated by primary endodontic treatment (41.38%) and in 49 of 60 teeth treated by nonsurgical retreatment (81.67%). The supplementary unadjusted Chi-Square analysis yielded a similar descriptive pattern (nominal *p* < 0.0005, Cohen’s w = 0.407).

The NiTi instrumentation system showed no evidence of an overall association with complete clinico-radiographic healing in the cluster-adjusted GEE analysis (Wald χ^2^(2) = 0.959, nominal *p* = 0.619). With VDW.ROTATE as the reference category, the OR was 1.422 for RECIPROC blue R25 (95% CI 0.494–4.098, nominal *p* = 0.514) and 2.180 for ProTaper Ultimate (95% CI 0.387–12.267, nominal *p* = 0.377). The corresponding supplementary unadjusted analysis yielded a nominal *p* value of 0.289 and Cohen’s w = 0.167.

A secondary exploratory analysis according to the type of NiTi motion also showed no evidence of an association with complete clinico-radiographic healing in the cluster-adjusted GEE model. Compared with reciprocating instrumentation, continuous rotation yielded an OR of 0.856 (95% CI 0.297–2.469, nominal *p* = 0.774). The corresponding supplementary unadjusted Pearson’s Chi-Square analysis yielded a nominal *p* value of 0.743.

Quadrant showed no evidence of an overall association with complete clinico-radiographic healing in the cluster-adjusted GEE analysis (Wald χ^2^(3) = 0.404, nominal *p* = 0.939). None of the quadrant-specific contrasts provided evidence of a difference from the Q2 reference category (Table 2). The supplementary unadjusted analysis similarly showed little evidence of a difference according to quadrant (nominal *p* = 0.935, Cohen’s w = 0.069).

Posterior teeth showed higher odds of complete clinico-radiographic healing than anterior teeth in the exploratory cluster-adjusted GEE analysis (OR = 6.625, 95% CI 1.91–23.01, nominal *p* = 0.003). Complete healing was observed in 8 of 22 anterior teeth (36.36%) and in 53 of 67 posterior teeth (79.10%). The corresponding supplementary unadjusted analysis yielded nominal *p* < 0.0005 and Cohen’s w = 0.397. Multi-rooted teeth also showed higher odds of complete healing than single-rooted teeth (OR = 3.536, 95% CI 1.166–10.718, nominal *p* = 0.026). Complete healing was observed in 28 of 49 single-rooted teeth (57.14%) and in 33 of 40 multi-rooted teeth (82.50%); the supplementary unadjusted analysis yielded nominal *p* = 0.010 and Cohen’s w = 0.272.

Because no adjustment for multiple exploratory comparisons was applied, all *p* values reported in these subgroup analyses are nominal and should be interpreted descriptively rather than as confirmatory evidence of independent effects.

### 3.2. Treatment Modality Analysis

Treatment modality was markedly imbalanced across the three NiTi instrumentation systems. VDW.ROTATE was used most frequently in primary-treatment cases, with 23 of 42 VDW.ROTATE-treated teeth (54.76%) belonging to the primary-treatment group. By contrast, 31 of 34 RECIPROC blue R25-treated teeth (91.18%) and 10 of 13 ProTaper Ultimate-treated teeth (76.92%) belonged to the nonsurgical retreatment group.

In the cluster-adjusted exploratory GEE analysis with nonsurgical retreatment coded as the outcome, NiTi instrumentation system showed an overall association with treatment modality (Wald χ^2^(2) = 12.815, nominal *p* = 0.002). With VDW.ROTATE as the reference category, RECIPROC blue R25 was associated with substantially higher odds of being used in nonsurgical retreatment cases (OR = 12.509, 95% CI 3.018–51.846, nominal *p* < 0.0005). The estimate for ProTaper Ultimate was also above unity but imprecise (OR = 4.035, 95% CI 0.780–20.886, nominal *p* = 0.096). The supplementary unadjusted analysis likewise showed a marked imbalance between treatment modality and NiTi instrumentation system (nominal *p* < 0.0005, Cohen’s w = 0.458) (Table 3).

Quadrant showed no evidence of an overall association with treatment modality in the cluster-adjusted GEE analysis (Wald χ^2^(3) = 2.415, nominal *p* = 0.491). None of the individual quadrant contrasts with Q2 as the reference category showed evidence of an association (Table 3). The supplementary unadjusted analysis similarly yielded nominal *p* = 0.388 and Cohen’s w = 0.184 (Table 3).

Treatment modality was also distributed unevenly according to tooth morphology. Posterior teeth showed higher odds of belonging to the nonsurgical retreatment group than anterior teeth (OR = 8.112, 95% CI 2.284–28.815, nominal *p* = 0.001). Primary treatment was recorded in 15 of 22 anterior teeth (68.18%) but in only 14 of 67 posterior teeth (20.90%). Multi-rooted teeth also showed higher odds of belonging to the retreatment group than single-rooted teeth (OR = 3.841, 95% CI 1.417–10.410, nominal *p* = 0.008). Nonsurgical retreatment was performed in 33 of 40 multi-rooted teeth (82.50%) compared with 27 of 49 single-rooted teeth (55.10%). The supplementary unadjusted analysis for root morphology yielded nominal *p* = 0.006 and Cohen’s w = 0.291 (Table 3).

These distributions indicate marked imbalances in instrumentation system selection and tooth morphology according to treatment modality in this cohort. Accordingly, the observed subgroup differences should be interpreted descriptively and should not be considered evidence of independent effects of treatment modality, instrumentation system, or anatomical characteristics.

### 3.3. Distribution According to NiTi Instrumentation System

Associations between the NiTi instrumentation system and the other variables included in the study were analyzed using Pearson’s chi-square tests, with Cohen’s w used to quantify effect size. Because some contingency tables included small cell counts, these results were interpreted cautiously and considered exploratory. The statistical results are presented in Table 4.

Descriptive differences in NiTi instrumentation system distribution were observed according to tooth type (nominal *p* < 0.0005, Cohen’s w = 0.460) and quadrant (nominal *p* = 0.035, Cohen’s w = 0.390), whereas the corresponding nominal *p* value for root morphology was 0.116 (Cohen’s w = 0.220). RECIPROC blue R25 was used only in posterior teeth, whereas anterior teeth were treated predominantly with VDW.ROTATE. Because these contingency-table comparisons were exploratory and no multiplicity adjustment was applied, the *p* values should be interpreted descriptively rather than as confirmatory evidence of associations (Table 4).

An additional exploratory analysis was performed to examine the non-random distribution of NiTi instrumentation systems according to the studied tooth-related characteristics. NiTi instrumentation system was treated as a three-category nominal outcome and analyzed using multinomial logistic regression with GEE, with VDW.ROTATE as the reference category. Patient identification was used as the clustering variable, and robust standard errors with an exchangeable correlation structure were applied. The model generated two logit comparisons: RECIPROC blue R25 versus VDW.ROTATE and ProTaper Ultimate versus VDW.ROTATE (Table 5).

The exploratory multinomial GEE analysis indicated differences in the distribution of NiTi instrumentation systems according to some tooth-related characteristics. However, several coefficients were non-estimable because of quasi-complete separation, and the estimates should therefore be interpreted cautiously.

Because the overall robust Wald test for quadrant was non-estimable, the quadrant-specific estimates are interpreted descriptively. In the RECIPROC blue R25 versus VDW.ROTATE comparison, the Q1 versus Q2 coefficient corresponded to higher estimated odds of RECIPROC blue R25 use (OR = 6.045, 95% CI 1.067–34.265, nominal *p* = 0.042). Multi-rooted morphology yielded higher estimated odds of RECIPROC blue R25 use compared with single-rooted morphology (OR = 2.571, 95% CI 1.010–6.546, nominal *p* = 0.048).

No corresponding nominal associations were observed in the ProTaper Ultimate versus VDW.ROTATE comparison. Given the sparse subgroup structure, non-estimable coefficients, wide confidence intervals, and absence of adjustment for multiple exploratory comparisons, these findings should be regarded as descriptive and hypothesis-generating.

## 4. Discussion

The principal finding of the present study was that complete clinico-radiographic healing at 36 months was observed in 68.54% of the analyzed teeth. Exploratory cluster-adjusted subgroup analyses yielded nominal differences according to treatment modality, tooth type, and number of roots; however, no adjustment for multiple testing was applied, and these findings cannot establish independent effects of these variables. The exploratory analyses showed no evidence of an association with the NiTi instrumentation system used or the type of NiTi motion. Because CBCT/Diagnocat documentation was available only for a subset of teeth and could contribute additional information beyond periapical radiography, differential outcome ascertainment cannot be excluded. Diagnocat availability was therefore not analyzed as an explanatory factor for final apical status.

These observations should be regarded as hypothesis-generating. Moreover, the instrumentation system was not randomly allocated, and its selection was related to treatment modality and case characteristics. Consequently, no independent or causal effect of a specific NiTi system can be inferred from these data.

The proportion of complete clinico-radiographic healing observed in our study, 68.54%, should be interpreted specifically in relation to the stringent outcome definition used. It should not be considered equivalent to a general treatment success rate. Published outcome estimates vary substantially according to whether strict or more permissive criteria are applied. For example, a radiographic study published in 2025 reported 68% success using strict criteria and 83% using loose criteria [14], illustrating the effect of outcome definition on the reported estimate. Broader healing or success estimates reported in systematic reviews and clinical trials [12,15] are therefore not directly comparable with the present 68.54% complete-healing proportion when different outcome definitions or follow-up intervals were used.

The interpretation of periapical healing should also take into account the duration of follow-up. Recent long-term data on nonsurgical endodontic retreatment suggest that radiographic healing and treatment success may improve over time, while tooth survival remains favorable during long-term observation. Therefore, differences between studies may reflect not only case mix and treatment protocols, but also the timing and strictness of outcome assessment [16,17,18,19].

In the present study, this issue was addressed by using a uniform 36-month follow-up point for all included teeth.

The exploratory analyses showed no evidence of an association between the NiTi instrumentation system used and complete clinico-radiographic healing. This finding should not be interpreted as evidence of equivalence between systems. The instrumentation system was not randomly allocated, and its use was imbalanced according to treatment modality, with RECIPROC blue R25 being used predominantly in retreatment cases and VDW.ROTATE more frequently in primary treatment cases.

The secondary analysis according to type of motion, reciprocating versus continuous rotation, showed no evidence of an association with complete clinico-radiographic healing. Because all included teeth were treated with engine-driven NiTi systems, the present findings cannot be extrapolated to manual instrumentation, and direct comparisons with manual techniques are outside the scope of this study [20,21,22].

Mechanical instrumentation should be interpreted as part of a broader therapeutic approach to apical periodontitis, not as an isolated determinant of periapical healing. NiTi systems may influence canal shaping, trajectory maintenance, and irrigant access, but the final biological effect also depends on intracanal infection control, irrigation and disinfection protocols, root filling materials and techniques, coronal sealing, and the overall treatment strategy. Therefore, the lack of evidence of an association between the NiTi system and final apical status should be interpreted within a broader therapeutic framework, not as an isolated effect of instrument design [9,10,23,24].

More importantly for the interpretation of the present cohort, instrumentation system selection was closely linked to treatment modality and tooth characteristics, making isolated attribution of the observed healing differences to a particular technical factor inappropriate.

An apparently paradoxical finding of our study was the higher proportion of teeth meeting the complete clinico-radiographic healing criteria in the nonsurgical retreatment subgroup (81.67%) compared with the primary-treatment subgroup (41.38%). This finding should not be interpreted as evidence that nonsurgical retreatment is intrinsically superior to primary treatment. The two subgroups were unequal in size (29 primary-treatment versus 60 retreatment cases), and the smaller primary-treatment subgroup reduced the precision of the corresponding healing estimate. In addition, the groups differed in case composition, while treatment modality was determined by previous endodontic status and clinical indication rather than by random allocation. Moreover, several relevant baseline prognostic variables, including lesion characteristics, technical quality, and coronal restoration quality, were unavailable or insufficiently standardized for adjustment. The observed difference may therefore reflect confounding by indication, case-selection characteristics, and unmeasured baseline differences rather than a genuine treatment effect. Accordingly, this subgroup finding should be regarded as exploratory and hypothesis-generating and requires confirmation in larger prospective studies with balanced groups and standardized assessment of relevant prognostic variables [25,26,27,28,29,30,31].

In the exploratory cluster-adjusted GEE analysis, posterior teeth showed higher odds of complete clinico-radiographic healing than anterior teeth, an unexpected finding that should be interpreted with caution in view of the imbalanced case distribution and potential residual confounding. Classical clinical studies and systematic reviews have shown that endodontic prognosis is influenced by multiple factors, including the initial periapical status, the quality of endodontic treatment, root canal filling, and coronal restoration, while tooth type or root complexity may interact with these factors [3,4]. In more recent literature, findings are not fully consistent. The cohort study published by Jurič et al. associated unfavorable outcomes with more complex anatomy, including premolars or molars and cases with two canals in a single root [6]. By contrast, the radiographic study published in 2025 identified an unfavorable effect of tooth type, including anterior teeth and premolars [14]. This variability may be partly explained by differences between clinical studies in outcome definition, follow-up duration, success criteria, and the prognostic factors analyzed, especially in studies including both endodontic treatment and nonsurgical retreatment [32,33]. The observed differences according to tooth type and number of roots may therefore reflect baseline lesion severity, treatment strategy, and the imbalanced distribution of cases across subgroups. Moreover, broad classifications based on tooth type or number of roots cannot capture the full variability of internal root anatomy. CBCT-based morphometric studies have documented substantial variation in dentin thickness within mandibular first molars, illustrating the anatomical detail that may be obscured when teeth are grouped into simplified morphological categories [34].

An important limitation concerns the ascertainment of final apical status. CBCT/Diagnocat documentation was available only for a subset of teeth and, when present, could contribute information beyond that provided by the periapical radiograph.

Because CBCT may identify persistent apical pathology that is not evident on two-dimensional radiographs, the sensitivity of outcome ascertainment may therefore have differed between teeth with and without additional CBCT documentation. A periapical-radiograph-only endpoint could not be reconstructed retrospectively for all cases independently of the CBCT/Diagnocat findings. Diagnocat availability was consequently not analyzed as an explanatory factor for final apical status.

This issue is consistent with recent CBCT-based literature suggesting that three-dimensional assessment may influence both the estimation of healing rates and the identification of prognostic factors, compared with studies based exclusively on periapical radiography. Therefore, direct comparison of success rates between studies should be made with caution, depending on the imaging method used for outcome assessment [26,35]. In addition, surveys of endodontists indicate that CBCT is increasingly integrated into endodontic practice [36]. However, image interpretation may also be influenced by acquisition and reconstruction characteristics and by artifacts generated by endodontic and restorative materials. Recent experimental evidence showed that activation of a metal artifact reduction algorithm did not consistently reduce such artifacts, emphasizing the importance of considering technical imaging conditions when interpreting CBCT-based endodontic findings [37].

This interpretation is also consistent with recent CBCT-based evidence suggesting that endodontic outcome is influenced by multiple interacting factors, including tooth type, clinician-related variables, apical preparation size and taper, irrigant strategy, and obturation technique. In the present study, the effect of the NiTi instrumentation system could not be estimated independently because system selection was non-random and was related to treatment modality and case characteristics. The present data therefore do not support attribution of the 36-month outcome to the isolated choice of a specific NiTi system [38,39,40,41,42,43,44,45,46].

Recent clinical evidence further supports interpreting periapical healing in relation to the complete treatment pathway. Randomized and prospective studies have reported favorable radiographic outcomes after different irrigation, foraminal-cleaning, and obturation protocols [47,48,49]. CBCT-based data also indicate that the extent and rate of osseous repair may vary according to baseline lesion characteristics and other clinical or treatment-related factors [50]. Additional prospective and randomized studies have reported favorable outcomes with different sealer and obturation protocols [51,52,53,54]. Longer-term studies extending to three to five years have reported favorable outcomes with calcium silicate-based sealer protocols, further supporting the need to distinguish the effect of the overall therapeutic strategy from that of any single instrumentation system [51,53,55].

The findings should be interpreted in light of the retrospective design and the dependence on the completeness of the available clinical and imaging records. Radiographs were interpreted during routine care by the treating endodontist, with senior review of uncertain cases. Several established prognostic variables could not be included in the present analysis. These included age, systemic conditions, smoking status, periodontal status, baseline lesion size or periapical index score, pulpal diagnosis, number of root canals, baseline technical quality, root filling quality, coronal restoration quality, and procedural complications. Some of these variables were absent from the retrospective records, whereas others were not documented with sufficient consistency or standardization for reliable case-level analysis. Their omission leaves a substantial risk of residual confounding and limits interpretation of the observed subgroup differences. In addition, eligibility required complete baseline, treatment, and 36-month follow-up documentation. Consequently, the analyzed cohort may differ systematically from cases excluded because of incomplete clinical or baseline imaging records, and selection bias cannot be excluded. Because the retrospective information available for the excluded cases was incomplete and insufficiently standardized, a meaningful comparison between included and excluded cases could not be performed, and the direction and magnitude of this potential selection bias could not be quantified. Because all procedures were performed by the same experienced endodontic specialist, clinician-related variability could not be assessed. Although this reduced interoperator heterogeneity, the observed outcomes may partly reflect operator-specific clinical performance, thereby introducing potential operator-related bias and limiting the generalizability of the findings to other clinical settings and clinicians with different levels of experience. Moreover, with only 28 not-healed outcomes, simultaneous inclusion of these additional variables in a prognostic model of the 36-month healing outcome would have produced a severely overparameterized and unstable model.

NiTi system selection was not randomized and was related to treatment modality and case characteristics. The primary-treatment and retreatment subgroups were also unequal in size (29 versus 60 teeth) and differed in their observed case composition. In the absence of random allocation and comprehensive baseline prognostic information, comparability between treatment modalities could not be established; therefore, the observed difference in complete healing should be interpreted descriptively rather than as a treatment effect. Similarly, the potential effect of the instrumentation system cannot be separated from treatment indication, tooth type, root morphology, and other unmeasured factors. Because CBCT/Diagnocat findings could contribute to outcome classification only when this additional imaging documentation was available, the sensitivity of outcome ascertainment was potentially different across the cohort. This creates a risk of differential detection bias and prevents Diagnocat availability from being interpreted or analyzed as an ordinary explanatory factor.

Some patients contributed more than one tooth. Within-patient correlation was therefore accounted for in the principal inferential analyses using cluster-adjusted GEE models. Nevertheless, the modest sample size and the limited number of not healed outcomes reduced the precision of the resulting estimates. With only 28 not-healed outcomes, the dataset was not considered sufficient to support a stable multivariable prognostic model of the 36-month healing outcome that also adequately accounted for patient-level clustering. The study size was not determined by an a priori power calculation because all consecutive eligible cases identified during the predefined treatment period were included. The post hoc analysis indicated high achieved power for some of the observed associations but substantially lower power for others, particularly those involving NiTi system, quadrant, and type of NiTi motion. Consequently, nonsignificant findings should not be interpreted as evidence of equivalence or absence of an association. In addition, several exploratory subgroup comparisons were performed without adjustment for multiplicity. The resulting *p* values are therefore nominal and should be regarded as descriptive rather than confirmatory.

Study strengths include the predefined binary outcome criteria and the uniform 36-month follow-up time point.

Furthermore, the retrospective records did not permit complete verification of the radiographic acquisition parameters and projection geometry for every examination. The absence of study-specific examiner calibration, blinded radiographic reassessment, quantitative lesion measurements, and formal interobserver agreement represents an additional limitation. The stringent binary endpoint also has an interpretative limitation. Teeth were classified as healed only when complete clinical and radiographic resolution was documented. Consequently, the 28 teeth classified as not healed may represent heterogeneous biological states, including ongoing but incomplete osseous healing as well as persistent disease. The observed 68.54% should therefore be interpreted as the proportion achieving complete clinico-radiographic healing at 36 months rather than as an overall treatment success rate.

Future studies should use prospectively defined and standardized data collection, larger samples, and an adequate number of outcome events to evaluate these clinical, biological, and technical prognostic variables simultaneously.

### Clinical Relevance of the Study

This study provides 36-month clinical and radiographic data on final apical status after primary endodontic treatment and nonsurgical retreatment performed with engine-driven NiTi instrumentation. The findings emphasize that endodontic outcomes should be interpreted through comprehensive case assessment, including treatment indication, tooth morphology, the method of outcome ascertainment, and the overall therapeutic protocol, rather than through any single technical or clinical variable.

The observed subgroup differences were derived from exploratory cluster-adjusted univariable analyses and should not be interpreted as independent prognostic effects. Prospective studies with balanced treatment groups, standardized imaging protocols, and adequately powered multivariable analyses are required to confirm whether any of these variables have prognostic relevance.

## 5. Conclusions

Within the limits of this retrospective study, complete clinico-radiographic healing at 36 months was observed in 68.54% of the analyzed teeth. The remaining 31.46% did not meet the stringent complete-healing criteria and should not be interpreted uniformly as representing persistent disease or treatment failure. Exploratory cluster-adjusted subgroup comparisons showed higher odds of complete healing in nonsurgical retreatment than in primary treatment, in posterior than in anterior teeth, and in multi-rooted than in single-rooted teeth. Because no adjustment for multiple testing was applied and important measured and unmeasured confounding remained, these findings should not be interpreted as independent prognostic effects. The principal contribution of the study is therefore the descriptive 36-month complete-healing outcome rather than attribution of that outcome to individual technical or anatomical factors. Because CBCT/Diagnocat information could contribute to outcome classification only in a subset of teeth, differential detection bias cannot be excluded.

## Figures and Tables

**Table 1:** Tooth-level descriptive characteristics of the analyzed sample (*N* = 89 teeth).

Parameter	Value	Prevalence	95% Wilson CI
Patient gender, represented at tooth level	Female	46 (51.69%)	41.45–61.78%
	Male	43 (48.31%)	38.22–58.55%
Final apical status	Complete clinico-radiographic healing	61 (68.54%)	58.30–77.25%
	Did not meet complete-healing criteria	28 (31.46%)	22.75–41.70%
Treatment modality	Primary treatment	29 (32.58%)	23.74–42.87%
	Nonsurgical retreatment	60 (67.42%)	57.13–76.26%
Diagnocat-based imaging documentation	Available	74 (83.15%)	74.04–89.51%
	Not available	15 (16.85%)	10.49–25.96%
NiTi instrumentation system	RECIPROC blue R25	34 (38.2%)	28.79–48.59%
	ProTaper Ultimate	13 (14.61%)	8.74–23.40%
	VDW.ROTATE	42 (47.19%)	37.15–57.46%
Quadrant	Q1	9 (10.11%)	5.41–18.11%
	Q2	38 (42.7%)	32.93–53.07%
	Q3	24 (26.97%)	18.84–37.00%
	Q4	18 (20.22%)	13.19–29.72%
Tooth type	Anterior	22 (24.72%)	16.93–34.6%
	Posterior	67 (75.28%)	65.4–83.07%
Number of roots/root morphology	Single-rooted	49 (55.06%)	44.73–64.97%
	Multi-rooted	40 (44.94%)	35.03–55.27%

Data are presented as n (%) with 95% Wilson confidence intervals. Gender is a patient-level characteristic. The gender counts shown in this table represent teeth categorized according to the gender of the corresponding patient and therefore sum to 89 teeth rather than 69 patients.

**Table 2:** Exploratory cluster-adjusted univariable GEE associations between selected study variables and 36-month final apical status.

Parameter	Value	Complete Clinico-Radiographic Healing	Did Not Meet Complete-Healing Criteria	Total (*N* = 89)	OR for Complete Clinico-Radiographic Healing (95% CI)	*p* for OR	Overall Robust Wald χ^2^/*p*
		61 (68.54%)	28 (31.46%)				
Treatment modality	Primary treatment	12 (41.38%)	17 (58.62%)	29 (100%)	1	-	-
	Nonsurgical retreatment	49 (81.67%)	11 (18.33%)	60 (100%)	6.205 (2.088–18.439)	0.001	
NiTi instrumentation system	VDW.ROTATE	26 (61.9%)	16 (38.1%)	42 (100%)	1		0.959/ 0.619
	RECIPROC blue R25	24 (70.59%)	10 (29.41%)	34 (100%)	1.422 (0.494–4.098)	0.514	
	ProTaper Ultimate	11 (84.62%)	2 (15.38%)	13 (100%)	2.180 (0.387–12.267)	0.377	
Quadrant	Q2	26 (68.42%)	12 (31.58%)	38 (100%)	1		0.404/ 0.939
	Q1	7 (77.78%)	2 (22.22%)	9 (100%)	1.615 (0.312–8.370)	0.568	
	Q3	16 (66.67%)	8 (33.33%)	24 (100%)	0.908 (0.237–3.594)	0.908	
	Q4	12 (66.67%)	6 (33.33%)	18 (100%)	0.908 (0.238–3.575)	0.908	
Tooth type	Anterior	8 (36.36%)	14 (63.64%)	22 (100%)	1		-
	Posterior	53 (79.1%)	14 (20.9%)	67 (100%)	6.625 (1.908–23.005)	0.003	
Number of roots/root morphology	Single-rooted	28 (57.14%)	21 (42.86%)	49 (100%)	1		-
	Multi-rooted	33 (82.5%)	7 (17.5%)	40 (100%)	3.536 (1.166–10.718)	0.026	
Type of NiTi motion	Reciprocating	24 (70.59%)	10 (29.41%)	34 (100%)	1		-
	Continuous rotation	37 (67.27%)	18 (32.73%)	55 (100%)	0.856 (0.297–2.469)	0.774	

ORs and 95% CIs are derived from separate univariable logistic GEE models accounting for clustering of teeth within patients. The modeled outcome was complete clinico-radiographic healing (coded 1), whereas failure to meet the complete-healing criteria was coded 0. Reference categories were primary treatment for treatment modality, VDW.ROTATE for NiTi instrumentation system, Q2 for quadrant, anterior teeth for tooth type, single-rooted teeth for root morphology, and reciprocating motion for type of NiTi motion. Thus, OR > 1 indicates higher odds of complete clinico-radiographic healing relative to the specified reference category. Overall robust Wald tests are reported for multicategory factors. All *p* values are nominal because no adjustment for multiple exploratory comparisons was applied. The dash indicates that no overall robust Wald χ^2^ was computed for dichotomous variables.

**Table 3:** Exploratory cluster-adjusted univariable GEE associations between instrumentation and tooth-related characteristics and treatment modality.

Parameter	Value	Primary Treatment	Nonsurgical Retreatment	Total (*N* = 89)	OR for Nonsurgical Retreatment (95% CI)	*p* (OR)	Overall Robust Wald χ^2^/*p*
		29 (32.58%)	60 (67.42%)				
NiTi instrumentation system	VDW.ROTATE	23 (54.76%)	19 (45.24%)	42 (100%)	1		12.815/0.002
	RECIPROC blue R25	3 (8.82%)	31 (91.18%)	34 (100%)	12.509 (3.018–51.846)	<0.0005	
	ProTaper Ultimate	3 (23.08%)	10 (76.92%)	13 (100%)	4.035 (0.780–20.886)	0.096	
Quadrant	Q2	13 (34.21%)	25 (65.79%)	38 (100%)	1		2.415/ 0.491
	Q1	1 (11.11%)	8 (88.89%)	9 (100%)	4.160 (0.455–38.065)	0.207	
	Q3	10 (41.67%)	14 (58.33%)	24 (100%)	0.728 (0.200–2.644)	0.629	
	Q4	5 (27.78%)	13 (72.22%)	18 (100%)	1.352 (0.339–5.396)	0.669	
Tooth type	Anterior	15 (68.18%)	7 (31.82%)	22 (100%)	1		-
	Posterior	14 (20.90%)	53 (79.10%)	67 (100%)	8.112 (2.284–28.815)	0.001	
Number of roots/root morphology	Single-rooted	22 (44.90%)	27 (55.10%)	49 (100%)	1		-
	Multi-rooted	7 (17.50%)	33 (82.50%)	40 (100%)	3.841 (1.417–10.410)	0.008	

ORs and 95% CIs are derived from separate univariable logistic GEE models accounting for clustering of teeth within patients. The modeled outcome was nonsurgical retreatment (coded 1), whereas primary treatment was coded 0. Reference categories were VDW.ROTATE for NiTi instrumentation system, Q2 for quadrant, anterior teeth for tooth type, and single-rooted teeth for root morphology. Thus, OR > 1 indicates higher odds of belonging to the nonsurgical retreatment group relative to the specified reference category. Overall, robust Wald tests are reported for multicategory factors. All *p* values are nominal because no adjustment for multiple exploratory comparisons was applied.

**Table 4:** Distribution of tooth-related characteristics across NiTi instrumentation systems.

Parameter	Value	RECIPROC Blue R25, n (%)	ProTaper Ultimate, n (%)	VDW.ROTATE, n (%)	Total (*N* = 89)	Nominal Pearson’s χ^2^ *p*/Cohen’s w
		34 (38.2%)	13 (14.61%)	42 (47.19%)		
Quadrant	Q1	7 (77.78%)	0 (0%)	2 (22.22%)	9 (100%)	*p* = 0.035, Cohen’s w = 0.390
	Q2	11 (28.95%)	8 (21.05%)	19 (50.00%)	38 (100%)	
	Q3	10 (41.67%)	5 (20.83%)	9 (37.50%)	24 (100%)	
	Q4	6 (33.33%)	0 (0%)	12 (66.67%)	18 (100%)	
Tooth type	Anterior	0 (0%)	4 (18.18%)	18 (81.82%)	22 (100%)	*p* < 0.0005, Cohen’s w = 0.460
	Posterior	34 (50.75%)	9 (13.43%)	24 (35.82%)	67 (100%)	
Number of roots/root morphology	Single-rooted	14 (28.57%)	8 (16.33%)	27 (55.1%)	49 (100%)	*p* = 0.116, Cohen’s w = 0.220
	Multi-rooted	20 (50%)	5 (12.5%)	15 (37.5%)	40 (100%)	

**Table 5:** Exploratory cluster-adjusted multinomial logistic regression with GEE and robust standard errors of the association between NiTi instrumentation system and several tooth-related characteristics.

Parameter	Reference	Standard Error (Robust)	Coefficient Wald χ^2^	Nominal *p*	OR/CI 95%
**RECIPROC blue R25 vs. VDW.ROTATE (Reference)**					
Quadrant Q1	Q2	0.885	4.132	0.042	6.045 (1.067–34.265)
Quadrant Q3	Q2	0.750	0.755	0.385	1.919 (0.441–8.353)
Quadrant Q4	Q2	0.679	0.046	0.829	0.864 (0.228–3.274)
Posterior	Anterior	-	-	-	-
Multi-rooted morphology	Single-rooted	0.476	3.925	0.048	2.571 (1.010–6.546)
**ProTaper Ultimate vs. VDW.ROTATE (Reference)**					
Quadrant Q1	Q2	-	-	-	-
Quadrant Q3	Q2	0.699	0.063	0.802	1.267 (0.199–8.069)
Quadrant Q4	Q2	-	-	-	-
Posterior	Anterior	0.766	0.466	0.495	1.687 (0.375–7.585)
Multi-rooted morphology	Single-rooted	0.590	0.040	0.842	1.125 (0.354–3.577)

ORs and 95% CIs are derived from multinomial logistic regression with GEE models accounting for clustering of teeth within patients. The modeled outcome was NiTi instrumentation system (with VDW.ROTATE as reference). Other reference categories were Q2 for quadrant, anterior teeth for tooth type, single-rooted teeth for root morphology. Missing values represent non-estimable coefficients (due to a quasi-complete separation). All *p* values are nominal because no adjustment for multiple exploratory comparisons was applied. Because quasi-complete separation prevented estimation of a valid overall robust Wald test for the multicategory quadrant factor, no global quadrant test is reported; the quadrant-specific coefficient tests are presented descriptively only.

## Data Availability

The authors declare that the data from this research are available from the corresponding authors upon reasonable request.
